# Differential Effects of IFN-β on the Survival and Growth of Human Vascular Smooth Muscle and Endothelial Cells

**DOI:** 10.1089/biores.2014.0052

**Published:** 2015-01-01

**Authors:** Emiko Sano, Shinya Tashiro, Kouhei Tsumoto, Takuya Ueda

**Affiliations:** ^1^ Department of Medical Genome Sciences, Graduate School of Frontier Sciences, The University of Tokyo, Chiba, Japan.; ^2^ Department of Medical Proteomics Laboratory, Institute of Medical Science, The University of Tokyo, Tokyo, Japan.; ^3^ Department of Chemistry and Biotechnology, School of Engineering, The University of Tokyo, Tokyo, Japan.

**Keywords:** IFN-β, vascular smooth muscle cells, vascular endothelial cells, apoptosis, growth, p53 pathway

## Abstract

It has been documented that interferon (IFN)-β is effective against the genesis of atherosclerosis or hyperplastic arterial disease in animal model. The main mechanism of the efficacy was antiproliferative action on the growth of vascular smooth muscle cells (SMC). To understand more about the mechanisms that are responsible for the efficacy, we examined minutely the effects of IFN-β on the apoptosis and growth of vascular SMC and endothelial cells (EC). IFN-β enhanced SMC apoptosis in serum starved medium. Conversely, EC apoptosis induced by serum and growth factor deprivation was inhibited by IFN-β. The induction of SMC apoptosis and anti-apoptotic effect on EC linked to the expression of pro-apoptotic bax mRNA and caspase-3 activities. Anti-apoptotic bcl-2 mRNA was also up-regulated in EC. IFN-β inhibited SMC growth in a dose dependent manner. However, the growth of EC was rather enhanced by a low dose of IFNs. The antiproliferative effect on SMC associated with the activation of p21 and increase of G0/G1 arrested cells. The growth stimulation on EC was considered to link with increase of S and G2/M phase cells. SMC produced IFN-β in response to various stimulants. However, IFN-β was not induced in EC. These suggested that endogenous IFN-β from SMC may act on EC and affect to EC functions. In this study, it was clarified that IFN-β enhances SMC apoptosis and inhibits the EC apoptosis, and stimulates the EC growth. These effects were considered to contribute to a cure against hyperplastic arterial diseases as the mechanisms in the efficacy of IFN-β.

## Introduction

Recently, genome-wide expression analysis in patients with coronary artery stenosis and insufficient coronary collateral artery development was performed, and it was reported that interferon (IFN)-β signaling is enhanced in the patients.^1^ Thereafter, the efficacy of IFN-β on the vascular diseases was shown in a mouse model.^2^ However, the mechanisms related to the efficiency are not elucidated sufficiently, although antiproliferative effect against advanced smooth muscle cell (SMC) growth was shown.

To understand more about the mechanisms that are responsible for IFN-β induced efficacy, we intended to examine the IFN-β effects focusing on the apoptosis and growth of vascular SMC and endothelial cells (EC). These cells are functionally interrelated. However, the role and biological characteristics of these cells are quite different. Vascular EC can survive and proliferate depending on some growth factors such as vascular endothelial growth factor (VEGF)^3^ or basic fibroblast growth factor (bFGF)^4^ and contribute to the normal vascular conditions by producing many biologically active substances. Vascular SMC can proliferate in the serum supplemented medium without specific growth factors, although the growth is stimulated with various substances such as platelet derived growth factor (PDGF),^5^ bFGF,^6^ tumor necrosis factor-α (TNF-α),^7^ interleukin-1 (IL-1),^8^ and cytomegaro virus (CMV) infection.^9^ It has been documented that hyperplastic arterial diseases are triggered by the injury of EC caused by abruptly generation of oxygen radicals during the artery occlusion and recirculation.^10,11^ The proliferation of vascular SMC is a key event in the genesis of atherosclerosis or restenosis.^12^ To date, there are some reports related to the antiproliferative effect of IFN-β on SMC growth.^2,13^ However, only a few reports are documented about other mechanisms such as the effects on apoptosis or function of these cells.

Interferons (IFNs) are a family of related cytokines that mediate a wide range of diverse functions including antiviral, antiproliferative, antitumor, and immunomodulatory activities. IFNs are currently classified into two major groups of type 1 and type 2.^14^ Type 1 IFNs consist of several subtypes of IFN-α and a single IFN-β, as well as IFN-τ and IFN-ω. Type 2 IFN is designated IFN-γ, also known as immune IFN. These IFN types bind distinct cellular receptors and activate both individual and overlapping pathways. IFN-α and -β bind to the common receptor and have the homology of 50% in amino acid sequence.^15^ However, there are some reports about differential effects and transcriptional differences between IFN-α and IFN-β.^16,17^ IFN is known to give direct cytotoxic effects on primary malignant cells^18^ and induces apoptosis.^16,19–21^ Some molecular mechanisms have been clarified. IFN induced apoptosis linked to the activation of bax gene expression,^22,23^ caspase pathway,^24,25^ and TRAIL/Apo2L^16,26–28^ in human tumor cells. The bcl-2 family including anti-apoptotic bcl-2 and pro-apoptotic molecule bax plays a pivotal role in regulating apoptosis.^29^ Bcl-2 can prevent apoptosis by blocking the release of cytochrome c from mitochondria^30^ and inhibiting the activation of caspase-3.^31,32^ To date, 14 different caspases have been reported.^33^ Caspase-3, -6, and -7 are the major effector caspases that can cause proteolysis in apoptotic cells.^33–35^ Whereas the anti-apoptotic effect of IFN has been documented, IFN-α protected hepatocyte apoptosis during virus-infection,^36^ and IFN-β inhibited activated T-cell apoptosis.^37,38^ Recently, we reported that type 1 IFN inhibits the apoptosis of human vascular EC exposed to oxidative stress.^39^ These reports indicate differential effects of IFN on the apoptosis depending on the cell type. Recent molecular study has revealed that IFN-α/β signal affects the p53 responses in tumor suppression and antiviral defense.^40^ P53 inhibits cell cycle progression and has been known to be functionally inactivated in many human cancers.^41^ In mammalian cells, proliferation control is primarily achieved in the G1 phase of the cell cycle.^42,43^ IFN-α induced cell cycle arrest was associated with the upregulation of p21 and G1 arrest.^44^ It has been also demonstrated that p53 controls both the G2/M and G1 cell cycle checkpoints and mediates reversible growth arrest.^45^ Further, it was shown that both the apoptosis and growth inhibition by type 1 IFN correlate with activation of p21.^46,47^ It has been also documented that CMV infection causes advanced SMC proliferation by mutating p53 gene.^9^ These reports indicates that it is important to examine the status of p53 gene for the study relating to p53 pathway.

In this study, we could demonstrate the differential effects of IFN-β on the apoptosis and growth of vascular SMC and EC together with the related molecular mechanisms. These effects were considered to contribute to a cure against hyperplastic arterial diseases as the mechanisms in the efficacy of IFN-β.

## Materials and Methods

### Materials

Natural type of IFN-α (Sumitomo Pharmaceuticals, Osaka, Japan) and IFN-β (Toray Industries, Tokyo, Japan) and recombinant IFN-γ (Genentech, South San Francisco, CA) derived from *Escherichia coli* were used for experiments. Recombinant human PDGF-BB, IL-1β, TNF-α, and bFGF were purchased from Pepro Tech Inc. (Rocky Hill, NJ) and poly I/C was obtained from Yamasa Corporation (Chiba, Japan).

### Cell and cell culture

Human coronary arterial smooth muscle cells (HCASMC) and human aortic endothelial cells (HAEC) were purchased from Kurabo (Osaka, Japan). HCASMC were cultured routinely in Humedia-SB2 (Kurabosupplemented with 10% fetal calf serum (FCS, Life Technologies, Grand Island, NY) using plastic culture flasks (Corning, NY). HAEC were cultured routinely in M199 medium (Nissui Pharmaceutical, Tokyo, Japan) supplemented with 10% FCS and 10 ng/mL of bFGF (Pepro Tech, Inc.) using collagen coated culture flasks (Iwaki, Chiba, Japan).

### Measurement of dead cell number

The cells were cultured in 24 well plates (HCASMC) (Iwaki) or collagen coated 24 well plates (HAEC) (Iwaki) until confluent. The culture medium was replenished with serum and growth factor deprived medium. After the confirmation of cell death by staining with 0.45% trypan blue (Sigma-Aldrich, St. Louis, MO), floating cells detached from confluent culture were counted by coulter counter (Coulter counter Z1, Beckman Coulter, Fullerton, CA).

### Determination of apoptosis by flow cytometry

The apoptosis induced by serum and growth factor deprivation was analyzed by flow cytometry. The cells after treatment were harvested using trypsin-EDTA solution (Invitrogen, San Diego, CA) and fixed in ice-cold 70% ethanol over 30 min. After rinsing the cells with phosphate buffered saline [PBS](−) (Nissui Pharmaceutical, Tokyo, Japan) twice, the fixed cells were treated with 0.5% RNase A (Rosh Diagnostics, Indianapolis, IN) for one hour and added propidium iodide (PI) (Molecular Probes, Eugene, OR). The fluorescence detected with FL3 (610 nm) were measured using a FACS-Calibur flow cytometer (Becton Dickinson, Franklin Lakes NJ), and the DNA histogram was analyzed by Flowjo software (BioLegend, San Diego, CA).

### PCR–single strand conformation polymorphism analysis of p53 gene

The status of p53 gene in HCASMC was analyzed by PCR–single strand conformation polymorphism (PCR-SSCP) according to the previous report.^48^ The mutation of exons 5, 6, 7, and 8 of p53 gene was examined using rhodamine-labeled primers of these exons instead of multiplex PCR. PCR products were diluted 1:3 with formamide loading buffer and denatured at 95°C for 5min. The each sample of 5 μL was loaded onto a 6% nondenaturing acrylamide gel and electrophoresed for 5–7 h at 15°C. After electrophoresis, gels were analyzed using fluorescence imaging analyzer (FMBIO II Multi View, Takara, Tokyo Japan). The sequences of primer pairs for each exon are as follows. Exon 5, (Forward) 5′-CTGACTTTCAACTCTG-3′ and (Reverse) 5′-AGCCCTGTCGTCTCT-3′; exon 6, (F) 5′-CTCTGATTCCTCACTG-3′ and (R) 5′-CCAGAGACCCCAGTTGCAAACC-3′; exon 7, (F) 5′-TGCTTGCCACAGGTCT-3′ and (R) 5′-ACAGCAGGCCAGTGT-3′; exon 8, (F) 5′-AGGACCTGATTTCCTTAC-3′ and (R) 5′-TCTGAGGCATAACTGG-3′. As a control of mutation, following changed sequences in each exon were used. Exon 5, codon 143 GTG (Val) of wild type sequence to GCG (Ala); exon 6, codon 194 CTT (Leu) to TTT (Phe); exon 7, codon 245 GGC (Gly) to AGC (Ser); exon 8, codon 273 CGT (Arg) to CAT (His). These mutant fragments for each p53 exon were supplied by Takara.

### Confirmation of growth suppressor function of p53

The growth suppressor function of p53 was examined using antisense oligodeoxynucleotides. The cells (1×10^4^) were seeded in 24 well plates and cultured in the 2% FCS added medium together with oligodeoxynucreotides. The proliferated cells were counted by coulter counter. Two different phosphorothioate p53 and control oligodeoxynucleotides were prepared by Japan Bioservice (Saitama, Japan). The sequences of these anti-sense and scrambled control nucleotides are as follows. AS1: 5′-CCCTGCTCCCCCCTGGCTCC-3′ (772–779), which was synthesized according to the previous report.^49^ AS2: 5′-CGGCTCCTCCATGGCAGT-3′ (209–286), which was originally constructed in our laboratories. Control: 5′-CGGTGATCTCCAGAGTATGC-3′, which is a scrambled sequence.

### Isolation of total RNA and real time and semiquantitative RT-PCR analysis

Total RNA was extracted from cultured cells for real time RT-PCR analysis using RNeasy Mini Kit (Qiagen, Valencia, CA). After the determination of RNA concentration at the absorbance of OD 260 nm, RNA was reverse-transcribed to cDNA with SuperScript Reverse Transcriptase II (Invitrogen, Carlsbad, CA). Real time PCR was performed on the ABI 7900HT (Applied Biosystems, Tokyo, Japan) with SYBR green PCR mix (Takara-Bio, Mountain View, CA). On the other hand, semiquantitative RT-PCR method was carried out for the comparison of mRNA expression level by band intensity. Reversibly transcribed products were used for PCR reaction using GeneAmp 2400 PCR system (Applied Biosystems, Foster City, CA). The PCR reaction was carried out in pertinent cycle number determined by ensuring amplification in the linear range using 20, 25, 30, and 35 cycles experimentally. The amplified products of were electrophoresed on 2% agarose gel containing ethidium bromide. The gel was visualized by ultraviolet irradiation and the photograph was taken using a fluorescence imaging analyzer (FMBIO, Takara, Tokyo, Japan). The primer pairs used for RT-PCR were as follows. The sequences of the primers for IFN-β were created from the sequence in cording region based on the data base (GENBank no. NM002176); (F) 5′-AATTGCTCTCCTGTTGTGCTTCTCC-3′ and (R) 5′-TGACTGTAGTCCTTGGCCTTCAG-3′ with a product of 459 base pairs. The primers of p53 were purchased from Takara (Tokyo, Japan); (F) 5′-CTGGCCCCTCCTCAGCATCTTAT-3′ and (R) 5′-CTCGTGGTGAGGCTCCCCTTTCTT-3′ (333-bp product). The primer pair of p21 was synthesized according to the sequences reported by Abiko et al.^50^ (F) 5′-CCCAGTGGACAGCGAGCAGC-3′ and (R) 5′-TCCCCTGAGCGAGGCACAAG-3′ with a product of 306 bp. The primers of bax (412-bp product), bcl-2 (380-bp product), and β-actin (275-bp product) were used ApoPrimer Set (Bcl-2 family, Takara, Tokyo, Japan). In quantitative RT-PCR analysis, fold changes of relative mRNA expression were calculated using the comparative 2^−ΔΔCt^ method using β-actin to normalize mRNA level as previously described.^51^

### Determination of caspase-3 activity

Caspase-3 activities were analyzed by the spectrophotometric detection of the chromophore p-nitroaniline (pNA) generated from the cleavage of the labeled caspase-specific substrates (DEVD-pNA) by caspase-3 using ApoAlert caspase colorimetric assay kit (Clontech, Mountain View, CA). The cells were collected and lysed with lysis buffer for 10 min on ice. The cell lysate was centrifuged at 12,000 rpm for 15 min at 4°C. After the addition of reaction buffer, caspase-3 substrate was added and incubated at 37°Cfor 1.5 h. The absorbance at 380 nm was measured by NanoDrop-2000 (Thermo Scientific, Wilmington, DE). Caspase-3 inhibiter, DEVD-fmk was added into the fluid separated from cell debris. The activities were determined from the pNA calibration curve prepared according to the manufacture's protocol and expressed as fold increase against the value of control cells.

### Western blot analysis

The cells were lysed in the RIPA buffer (Takara-bio, Shiga, Japan). The cell lysates were centrifuged at 12,000 rpm for 10 min and the protein contents of the supernatants were measured by Bradford method. Each sample of 50 μg was loaded on 4 to 20% SDS-PAGE gel and transferred onto polyvinylidene difluoride membrane (Thermo Scientific, Rockford, IL). Transferred membranes were treated with mouse anti-human p53 antibody (MCA 1703, UK-Serotec Ltd., Oxford, UK). Thereafter, the membranes were treated with anti-mouse immunoglobin g–horseradish peroxidase (Santa Cruz Biotechnology, Santa Cruz, CA). The proteins were detected using ECL system (DuPont Pharmaceuticals, Boston, MA) and visualized using ImageQuant Las 4000 (GE Healthcare Japan, Tokyo, Japan). The band intensity was normalized by those of β-actin detected by the monoclonal antibody (Wako Pure Chemical, Osaka, Japan).

### Cell proliferation assay

The proliferation assay was carried out using 24-well plates (HCASMC) or collagen coated 24-well plates (HAEC). Ten thousand cells were seeded and precultured for 24 h before the treatment. The proliferated cells were harvested using trypsin–ethylenediaminetetraacetic acid (EDTA) solution (Invitrogen, San Diego, CA) and counted by Coulter counter.

### Cell cycle distribution analysis

The cells were cultured in respective growth medium for 24 h for the attachment, and IFN-β was added into fresh growth medium. The cells were harvested using trypsin–EDTA solution and rinsed with PBS(−). The cells were fixed in ice-cold 70% ethanol over 30 min and washed with PBS(−) twice. The fixed cells were treated with 0.5% RNase A for one hour and added PI. Then, their fluorescence detected with FL3 (610 nm) were measured using FACS-Calibur flow cytometer, and the DNA histogram was analyzed by Flowjo software.

### IFN-β production and quantification

Confluent cells cultured in each growth medium were stimulated with various stimulants in 2% FCS supplemented medium without growth factor. The supernatants were harvested at 48 h after the addition of stimulants for the determination of IFN-β contents. The quantification of IFN-β was carried out using an enzyme-linked immunosorbent assay (ELISA) kit developed by Toray Industries (Tokyo, Japan).

### Statistical analysis

All of the experiments related to cell counts were replicated over three times. Average cell number and standard error of the mean were calculated using Excel software. Appropriate comparisons were made by the Tukey-Kramer method for multiple comparisons using JMP software (Ver. 3, SAS Institute, Cary, NC). A value of *p*<0.05 was evaluated as statistically significant.

## Results

### Effects of IFN-β on the apoptosis

**FIG. 1.**
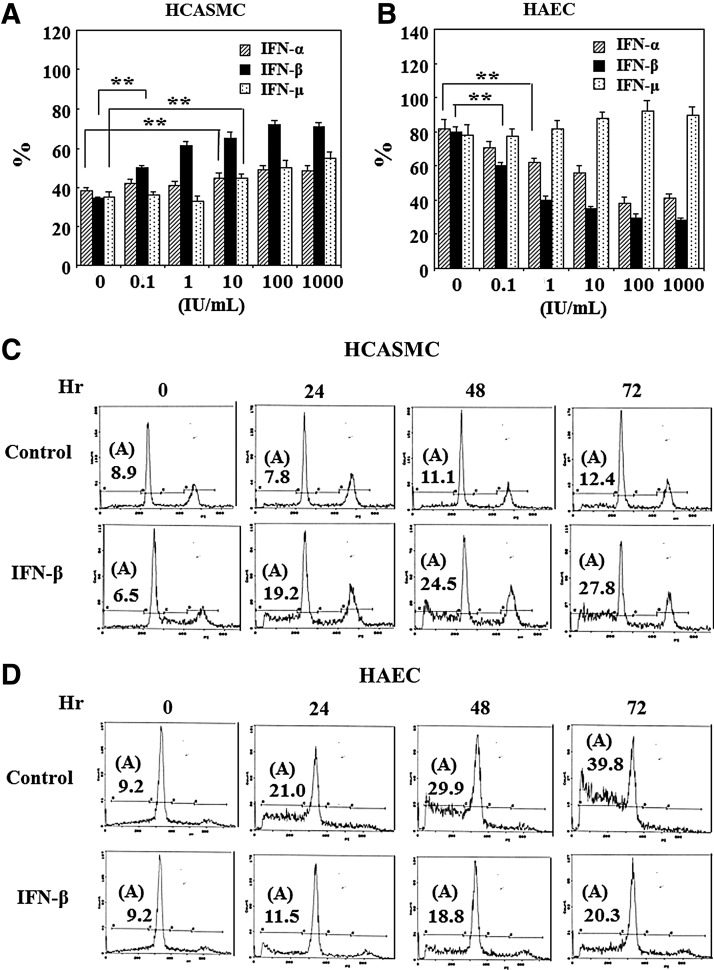
Effects of interferon beta (IFN-β) on the apoptosis. **(A)** Effects of IFNs on dead cell generation of human coronary arterial smooth muscle cell (HCASMC). Confluent cells grown in 24-well plates were treated with IFNs for 4 days in serum-starved medium. After the staining dead cells with 0.45% trypan-blue solution, the floating dead cells were counted by Coulter counter. The average cell number and the standard error (SE) of the mean in the four wells were calculated. The proportion (%) of dead cells was expressed as the ratio of dead cells against starting confluent cell number (1×10^5^ cells per well). **p<0.01, compared with each IFN minus and plus. **(B)** Effects of IFNs on dead cell generation of human aortic endothelial cell (HAEC). Confluent cells proliferated in 24 well collagen coated plates were treated with IFNs without serum and growth factors. The dead cells were counted after 4 days of IFN-β treatment. **p<0.01, compared with IFN-β minus and plus. **(C, D)** Apoptosis analysis by flow cytometry. The confluent HCASMC and HAEC were treated with 1,000 IU/mL of IFN-β. After the fixation, the cells were stained with propidium iodide (PI) solution and the fluorescence of the cells was analyzed by flow cytometer. The proportion (%) of apoptotic cells with degraded DNA was shown in the histogram as **(A)**.

At first, the effects of various IFNs on dead cell generation were examined. HCASMC and HAEC were cultured in each growth medium until confluent (1×10^5^ cells/well). IFNs were treated in the medium without serum and growth factor. The floating cells were stained with 0.45% trypan-blue solution for the confirmation of cell death and counted by coulter counter. As shown in Fig. 1A, the dead cells of HCASMC increased by IFN treatment in a dose dependent manner. IFN-β was most effective to HCASMC death. Whereas IFN-α and IFN-β inhibited dead cell generation of HAEC in a dose dependent manner as shown in Fig. 1B, IFN-γ did not inhibit the cell death. Next, the effects of IFN-β on the death of these cells were investigated by flow cytometry. The confluent cultures of HCASMC and HAEC were treated with IFN-β of 1,000 IU/mL in the serum and growth factor starved medium. The degraded DNA of apoptotic cells were detected in the histogram (Fig. 1C, D). The apoptotic cells of HCASMC increased with time elapse by IFN-β treatment as shown in Fig. 1C, whereas HAEC apoptotic cells decreased by IFN-β in spite of the vigorous apoptosis of control cells cultured in serum and growth factor starved medium, as shown in Fig. 1D. These results indicate the opposite function of IFN-β on the apoptosis of HCASMC and HAEC.

### p53 status and function of HCASMC

**FIG. 2.**
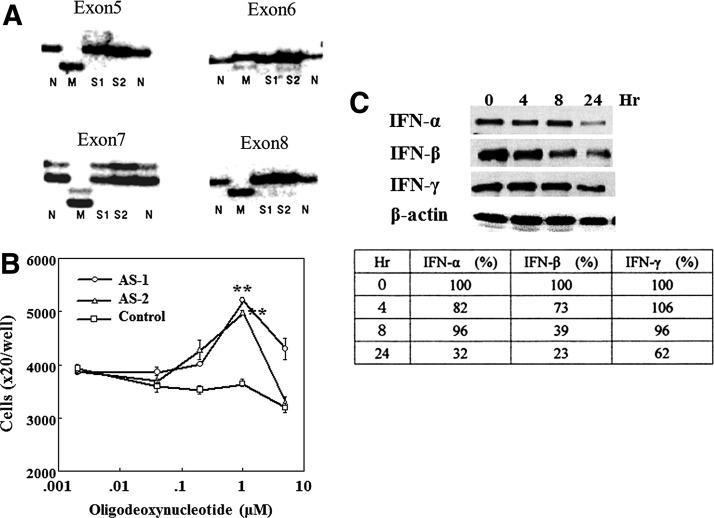
P53 status and function of HCASMC. **(A)** PCR–single-strand conformation polymorphism (PCR-SSCP) analysis of p53 gene. The cells were serially subcultured in the medium with or without 20 ng/mL platelet derived growth factor (PDGF)-BB. After preparation of genomic DNA from these cells, PCR and SSCP analysis were performed. PCR products of each exon were electrophoresed and visualized using fluorescent imaging analyzer. N, normal control; M, mutated control; S1, cell sample without PDGF-BB; S2, cell sample with PDGF-BB. **(B)** Growth suppressor function of p53. The cells of 1×10^4^ were seeded in 24 well plates. After the preculture for 24 h, the cells were proliferated in 2% fetal calf serum (FCS) supplemented medium together with two different p53 antisense (AS1, AS2) and control oligodeoxynucleotide. The proliferated cells were counted after 6 days using coulter counter. Average cell number in four wells and SE of the mean were demonstrated. **p<0.01, compared with control and p53 antisense addition. **(C)** Effects of IFNs on the p53 expression (Western blotting analysis). HCASMC were cultured until 80% confluent and treated with 1,000 IU/mL of IFN-α, -β, and -γ. The harvested cells were lysed with RIPA buffer and the extracts from the cells were analyzed for p53 contents. The proportion of band intensity normalized by these of β-actin is shown in the table.

The p53 gene status of HCASMC was examined using PCR-SSCP method to clarify the effects of IFN-β on the expression of p53 related genes since it has been documented that advanced vascular SMC growth occurs by p53 gene mutation. HCASMC were serially cultured for over a month in the presence or absence of PDGF-BB. After the preparation of genomic DNA, the mutations in exon 5, 6, 7, and 8 were analyzed by rhodamine labeled primers of these exons. PCR products were electrophoresed and visualized using fluorescent imaging analyzer. As shown in Fig. 2A, any obvious alterations in these exons were not found indicating the p53 is wild type. The growth suppressor function of the p53 was examined using two different p53 antisense oligodeoxynucleotides. The cells were cultured together with antisense and control nucleotides for 6 days. As shown in Fig. 2B, the growth of the cells was stimulated by the addition of p53 antisense nucleotides indicating the p53 of HCASMC possesses growth suppressor function. Next, the effects of IFNs on the p53 activation were examined by western blotting analysis. The cells were treated with 1,000 IU/mL of various IFNs. As shown in Fig. 2C, the expression of p53 were downregulated by these IFNs with time elapse. This suggested the activation of p53 by IFNs although the phospholylated p53 could not be detected by used anti-phospholylated p53 monoclonal antibody. The p53 protein level was most downregulated by IFN-β.

### Effects of IFN-β on the expression of p53 mediated genes

**FIG. 3.**
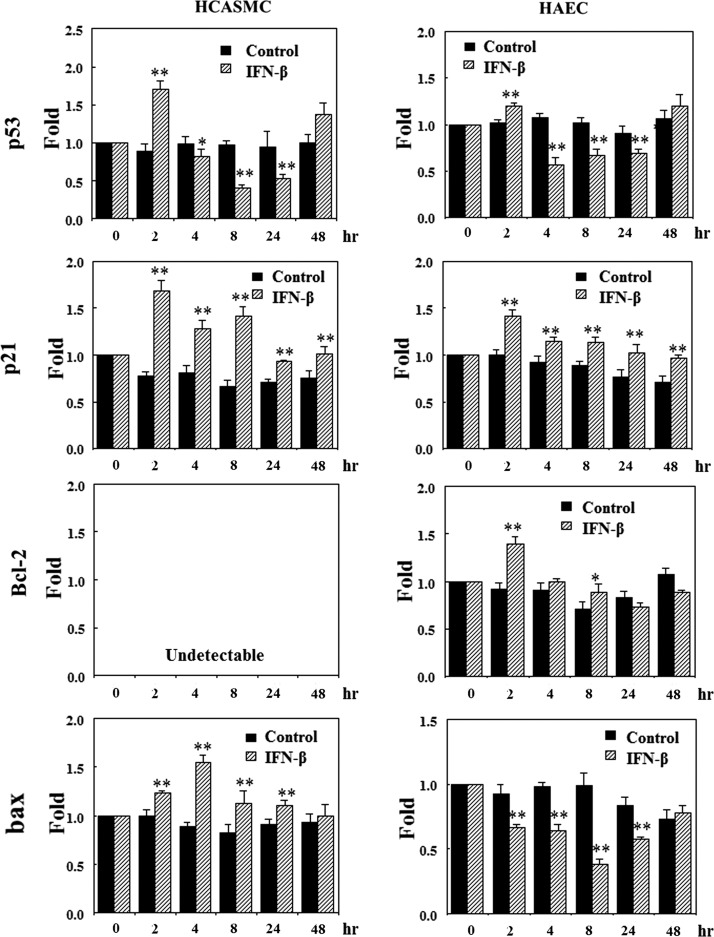
Effects of IFN-β on p53 related gene expression. HCASMC and HAEC were cultured until 80% confluent. The cells were treated with 1,000 IU/mL of IFN-β without serum and growth factors. Total RNA was isolated from harvested cells and expression level of mRNA was analyzed by real time reverse transcription polymerase chain reaction (RT-PCR). The fold changes of relative mRNA expression were calculated using the comparative 2^−ΔΔCt^ method using β-actin to normalize mRNA level as previously described.^50^ Experiments were performed three times independently. The results were expressed as the mean ± SE. **p < 0.01, compared with IFN-β minus control and plus.

The effects of IFN-β on the expression of p53, p21, bcl-2, and bax mRNA were investigated by real time RT-PCR method to understand the molecular mechanisms associated with apoptosis and growth of HCASMC and HAEC. The cells were cultured using each growth medium until 80% confluent. After IFN-β treatment at 1,000 IU/mL, the cells were harvested for the quantification of each mRNA. The results of RT-PCR analysis were shown in Fig. 3. The expression of p53 mRNA was upregulated in both the cells immediately after IFN-β treatment, and thereafter, the expression level decreased until 24 h, although it was recovered in 48 h. The expression pattern in HCASMC was similar to the result of western blotting (Fig. 2C) and suggested the activation of p53 by IFN-β. The expression of p21 mRNA in both the cells was upregulated by IFN-β, suggesting that it relates to the regulation of apoptosis and growth. The expression of bax mRNA in HCASMC was upregulated indicating apoptosis enhancement by IFN-β. On the contrary, the expression level was downregulated in HAEC. Anti-apoptotic bcl-2 mRNA was also upregulated in HAEC. Bcl-2 mRNA was not detected in HCASMC. These results indicate that IFN-β inhibits the HAEC apoptosis different from the case of HCASMC. These results demonstrate the differential effects of IFN-β on the expression of apoptosis related genes corresponding with the effects on apoptosis in HCASMC and HAEC.

### Effects of IFN-β on the caspase-3 activity

**FIG. 4.**
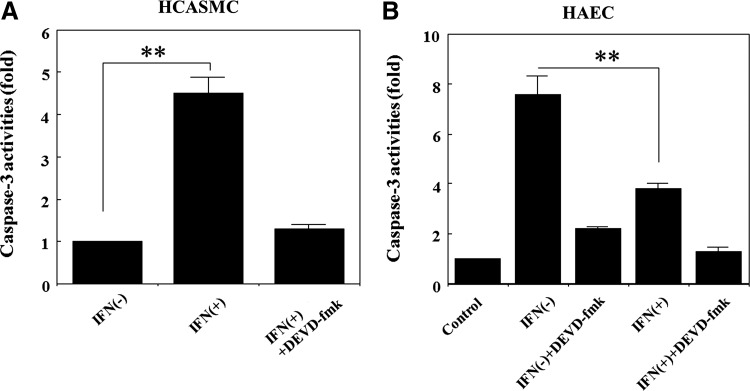
Effects of IFN-β on caspase-3 activity. **(A)** Effect on the activity of HCASMC. The 80% confluent cells were treated with 1,000 IU/mL of IFN-β for 48 h. The cells of 1×10^6^ were collected and lysed for the determination of caspase-3 activities. Caspase-3 activities were determined by the spectrophotometric detection of the p-nitrianiline (pNA) cleaved by caspase-3 from the labeled caspase-specific substrates (DEVD-pNA). DEVD-fmk, which is a caspase-3 inhibiter, was added into the extracts of IFN-β treated cells. **p<0.01, compared with IFN-β minus and plus. **(B)** Effect on the activity of HAEC. The cells cultured in collagen coated flasks were treated with 1,000 IU/mL of IFN-β for 48 h without serum and growth factors. The cells of 1×10^6^ were collected and lysed for the colorimetric assay. Caspase-3 inhibiter (DEVD-fmk) was added to both the cells of IFN-β (-) and IFN-β (+). The activity was shown as fold increase obtained from the comparison with the activity of the cells cultured in basic fibroblast growth factor (bFGF) (10 ng/mL) alone. **p<0.01, compared with IFN-β minus and plus.

The effects of IFN-β on the activation of caspase-3 were examined using a colorimetric assay. HCASMC and HAEC were cultured in each growth medium until confluent. The cells were treated with 1,000 IU/mL of IFN-β for 48 h in serum and growth factor deprived medium. The cells were harvested and the extracts isolated from 1×10^6^ cells were used for the analysis of caspase-3 activities. Caspase-3 inhibiter DEVD-fmk was added to the extracts for the confirmation of caspase-3 activities. As shown in Fig. 4A, IFN-β increased caspase-3 activity in HCASMC, indicating apoptosis enhancement, whereas the activity in HAEC was downregulated by IFN-β, demonstrating the anti-apoptotic effect of IFN-β, as shown in Fig. 4B. In the case of HAEC, the activity of the cells treated with bFGF alone was used as control. These results correlate with the results of dead cell generation (Fig. 1A, B) and flow cytometric apoptosis analysis (Fig. 1C, D).

### Effects of IFN-β on the proliferation

**FIG. 5.**
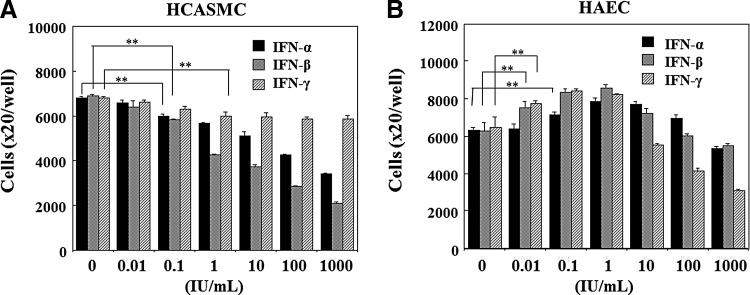
Effects of IFNs on the growth. **(A)** Effects of IFNs on HCASMC growth. The cells of 1×10^4^ were seeded in 24-well plates and were cultured in the growth medium. IFNs were added to fresh culture medium. The proliferated cells were counted at 5 days after IFN treatment by coulter counter. The average cell number and the SE in four wells are shown in the figure. **p<0.01, compared with each IFN minus and plus. **(B)** Effects of IFNs on HAEC growth. The cells were precultured in 24 well collagen coated plates for 24 h. IFNs were treated for 5 days in fresh growth medium. The average cell number and the SE in four wells are shown in the figure. **p<0.01, compared with each IFN minus and plus.

**FIG. 6.**
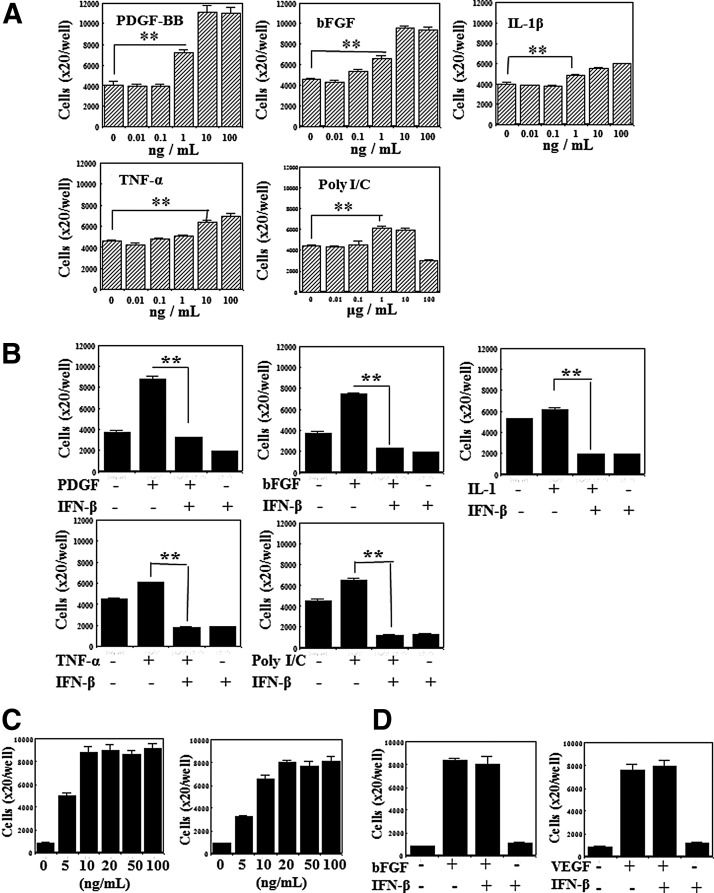
Antiproliferative effects of IFN-β. **(A)** HCASMC growth stimulation by various substances. The cells of 1×10^4^ were seeded in 24-well plates. After the preculture for 24 h, the cells were treated with PDGF-BB, bFGF, interleukin (IL)-1β and poly I/C treatments. After 5 days, the proliferated cells were counted using coulter counter. Average cell numbers and SEs of the mean obtained from four wells are shown in the figure. **p<0.01, compared with untreated and treated cells. **(B)** Effects of IFN-β on the HCASMC growth. After the preculture for 24 h, the cells were treated with PDGF-BB (10 ng/mL), bFGF (10 ng/mL), tumor necrosis factor (TNF)-α (20 ng/mL), IL-1β (20 ng/mL), and poly I:poly C (10 μg/mL) together with IFN-β of 100 IU/mL. The proliferated cells were counted after 4 days of the treatments. Average cell numbers with SEs in four wells were calculated. **p<0.01, compared with IFN-β minus and plus. **(C)** Growth factor–dependent HAEC growth. The cells of 2×10^4^ were cultured in 24-well collagen-coated plates in the presence of bFGF or vascular endothelial growth factor (VEGF) for 4 days. The proliferated cells were counted using coulter counter. Average cell numbers and SEs in four wells are shown. **p<0.01, compared with untreated and treated cells. **(D)** Effects of IFN-β on the HAEC growth. The cells were cultured in the medium supplemented with 10 ng/mL of bFGF or 20 ng/mL of VEGF respectively in the presence of 100 IU/mL of IFN-β. The proliferated cells were counted after 4 days. Average cell numbers and the SEs of the mean were shown.

At first, effects of various IFNs on the growth of HCASMC and HAEC were examined. After the preculture for attachment, these cells were proliferated using respective growth medium together with IFN-α, -β, and -γ for 5 days. The proliferated cells were counted using Coulter counter. The growth of HCASMC was inhibited by these IFNs in a dose dependent manner as shown in Fig. 5A. IFN-β was more effective than IFN-α and IFN-γ. The median effective dose (ED_50_) of IFN-β for the growth inhibition was about 10 IU/mL, which is extremely lower than those of IFN-α (3,850 IU/mL) or IFN-γ (over 1,000 IU/mL). The growth of HAEC was rather enhanced by these IFNs at low concentrations (under 10 IU/mL), as shown in Fig. 5B, although the growth was suppressed at high concentrations. Next, the effects of growth factor, cytokine, and double-stranded RNA on HCASMC growth were examined and the efficiency of IFN-β on the growth stimulated with these substances was investigated. PDGF-BB, bFGF, IL-1β, TNF-α, and poly I/C stimulated the HCASMC growth (Fig. 6A), however, IFN-β (at 100 IU/mL) inhibited all enhanced growth caused by these substances (Fig. 6B). HAEC could survive and proliferate depending on the specific growth factors, such as bFGF or VEGF (Fig. 6C). The treatment of over 10 ng/mL of bFGF or over 20 ng/mL of VEGF was necessary for getting maximum growth. As shown in Fig. 6D, the growth of HAEC in the presence of these growth factors was not inhibited by the addition of IFN-β (100 IU/mL).

### Effects of IFN-β on the cell cycle

**FIG. 7.**
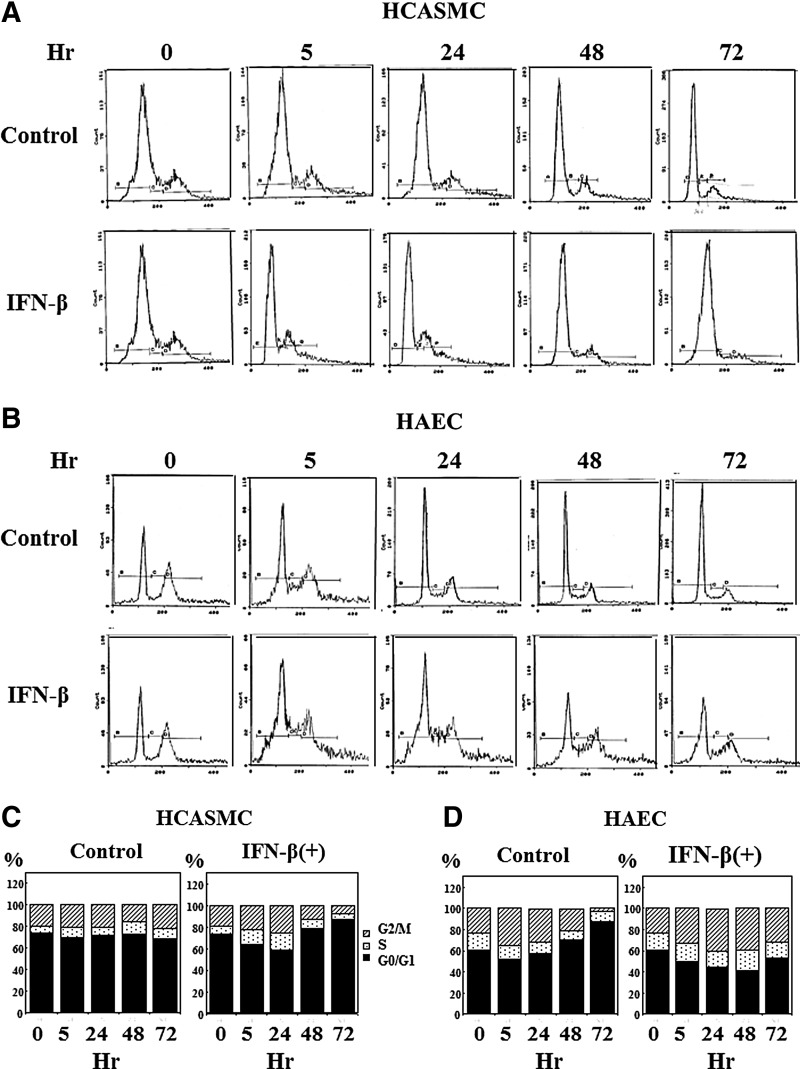
Effects of IFN-β on cell cycle. **(A)** Effects on HCASMC cell cycle. Growing cells were treated with 100 IU/mL of IFN-β for 5 to 72 h. The fixed cells were stained with PI for 30 min at 4°C and analyzed by flow cytometry. The DNA histogram was shown in the figure. **(B)** Effects on HAEC cell cycle. Growing cells were used for the analysis. The DNA histogram was shown in the figure. **(C)** Cell cycle distribution of HCASMC. The proportion of each cell cycle phase was shown based on the histogram of **(A)**. The proportion was calculated as the ratio to the total counts of G0/G1, S, and G2/M. **(D)** Cell cycle distribution of HAEC. The proportion of each cell cycle phase was calculated based on the histogram of **(B)** as the ratio to the total counts of G0/G1, S, and G2/M.

Logarithmically growing cells were used for the cell cycle distribution analysis. After the attachment of the cells, HCASMC and HAEC were proliferated in respective growth media together with 100 IU/mL of IFN-β. The cell cycle of these cells was analyzed by flow cytometry. The DNA histogram is shown in Fig. 7A and B. The proportion of each cell cycle phase was shown in Fig. 7C and D as the ratio to the total counts of G0/G1, S, and G2/M phase. IFN-β increased S and G2/M arrested cells in both the cells immediately after the treatment. Thereafter, the cell cycle of HCASMC arrested in G0/G1 correlating with the results of growth inhibition (Fig. 5A). The cell cycle distribution pattern of IFN-β treated HAEC did not alter during the cultivation, although most of the control cells arrested in G0/G1 suggesting growth arrest by contact inhibition as shown in Fig. 7B and D. These results indicate that the effects of IFN-β on the growth of HCASMC and HAEC are different.

### IFN-β production in HCASMC and HAEC

**FIG. 8.**
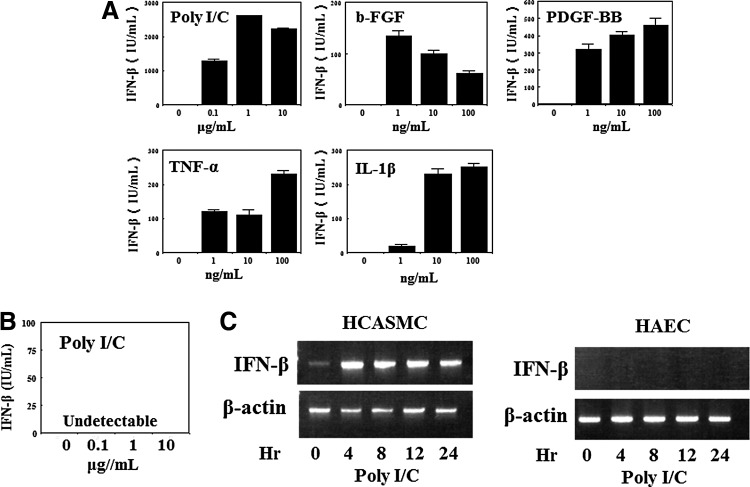
IFN-β production in HCASMC and HAEC. **(A)** IFN-β production in HCASMC. Two million cells in six-well plates were stimulated with poly I/C, bFGF, PDGF-BB, TNF-α, and IL-1β in the 2% FCS supplemented medium. After 48 h of the treatment, the supernatants were harvested for the determination of IFN-β contents by enzyme-linked immunosorbent assay. **(B)** IFN-β production in HAEC. The cells were cultured in collagen coated 6 well plates. The confluent cells were treated with poly I/C of 0.1 to 10 μg/mL in the medium supplemented with 2% FCS for 48 h. The IFN-β contents of culture fluids were analyzed. **(C)** The expression of IFN-β mRNA. HCASMC and HAEC were stimulated with poly I/C of 10 μg/mL and harvested at each time. Total RNA isolation and RT-PCR procedure were performed according to the method described in “Materials and Methods.” The amplified products of 5μL were loaded on 2% agarose gel and electrophoresed. The gel was visualized by ultraviolet irradiation.

IFN-β producing ability of HCASMC and HAEC was examined to clarify the role of IFN-β in hyperplastic arterial diseases. Confluent HCASMC cultures were treated with PDGF-BB, bFGF, IL-1β, TNF-α, and poly I/C, which are growth stimulating agents. The culture fluids were harvested at 48 h and IFN-β contents were determined using an ELISA kit with high sensitivity. As shown in Fig. 8A, HCASMC produced IFN-β in response to all of these stimulants, whereas IFN-β was not detected in the supernatants of HAEC cultures stimulated with poly I/C, which is a specific IFN-β inducer (Fig. 8B). To confirm these results, the expression of IFN-β mRNA by poly I/C was examined using semiquantitative RT-PCR. HCASMC and HAEC were stimulated with 10 μg/mL of poly I/C and the cells were harvested for the determination of IFN-β mRNA. As shown in Fig. 8C, the expression of IFN-β mRNA in HCASMC was enhanced by the stimulation of poly I/C; however, the expression was not detected in HAEC corresponding to the results in Fig. 8A, B.

## Discussion

IFNs have been documented to induce apoptosis in many tumor cells.^24,52,53^ The anti-apoptotic effects of IFN have been also documented.^36–39,54^ Type 1 IFN induced apoptosis linked with upregulation of bax gene expression,^22,23^ and activation of caspase-2 and caspase-3.^55^ The enhancement of HCASMC apoptosis by IFN-β was considered to be occurred by similar mechanisms to those in cancer cells. As was expected, IFN-β induced SMC apoptosis was associated with the enhancement of the bax gene expression (Fig. 3) and upregulation of caspase-3 activity (Fig. 4). On the contrary, these were downregulated and anti-apoptotic bcl-2 was upregulated in vascular EC (Figs. 3, 4), indicating the differential effects of IFN-β on the apoptosis and the related molecules of these cells. The expression of p21 mRNA was activated by IFN-β in both the cells (Fig. 3) as previously shown in other cells.^56^ Since p21 has been also known to relate to the apoptosis induction,^46,47^ the enhancement of p21 mRNA in HCASMC may affect the apoptosis induction. The up-regulation of p21 in HAEC (Fig. 3) may contribute to the apoptosis inhibition by arresting the cell cycle to G2/M as previously reported.^45^ These differential effects of IFN-β on the apoptosis of HCASMC and HAEC look like desirable actions for keeping normal condition of vascular vessels.

Molecular mechanisms of abnormal SMC proliferation generated in the hyperplastic arterial lesion have been documented on the basis of aberrant expression of p53 in SMC with CMV infection.^57^ It was shown that activated CMV could impair the growth suppressor function of p53 and contributes to the development of restenosis.^9^ It has been shown that 87% of the p53 gene mutations occur in exon 5 to exon 8 corresponding to the domain of the second to fifth of amino acid sequence.^58^ Further, the apoptosis induction by bax gene activation has been known to associate with the normal p53 status.^59^ Hence, p53 gene mutation was analyzed in this study to get the reliability of experiments relating to the activation of p53 pathway using PCR-SSCP method. As the result, any obvious mutations were not detected in these p53 exons of HCASMC (Fig. 2A).

The growth of HCASMC was inhibited by various types of IFNs similar to previous reports.^2,13,60^ In most cellular systems, the actions of IFN-α and IFN-β are generally the same, although IFN-β has been shown to be more potent and elicit IFN-β dependent gene activation at lower concentrations than IFN-α in several cell types.^16,25^ In our experiments, the antiproliferative effect of IFN-β was far better than those of other IFNs (Fig. 5). It was considered that the difference may be occurred by the different affinity to the receptor, since it has been documented that the difference of antiviral effects found in IFN-α subtypes correlates with the strength of binding to the receptor.^61^ There is a previous report about differential effects of IFN-β on the proliferation of vascular SMC and EC.^60^ We got similar results in this study (Fig. 5); however, we could clarified the related molecular mechanism and the cell cycle distribution pattern (Figs. 3, 7). The most common effect of IFN on cell cycle is G1 arrest although IFN affects different phases of the mitotic cycle in different cell systems.^62^ Although the proportion of S and G2/M phase cells increased in both the cells immediately after IFN-β treatment, the cell cycle of HCASMC arrested finally in G0/G1 (Fig. 7A, C). The upregulation of p21 in HCASMC was considered to link with the increase of G0/G1 arrested cells (Fig. 7A, C). The cell cycle distribution pattern of IFN-β treated EC did not alter during the cultivation, although the proportion of G0/G1 phase cells increased in untreated EC (Fig. 7B, D). This suggested proliferative action of IFNs on HAEC growth (Fig. 5B). The upregulation of p21 in HAEC did not associate with growth inhibition, although it may contribute to antiapoptotic effect.

It has been well known that IFN-β is an inducible protein different from IFN-α.^63^ HCASMC growth was stimulated with various stimulants existing in blood, and the cells produced IFN-β in response to these substances (Fig. 8A), whereas HAEC did not produce IFN-β, even the stimulation of poly I/C, which is a specific inducer for IFN-β (Fig. 8B). IFN-β mRNA was also not expressed in poly I/C stimulated HAEC, although the expression was enhanced in HCASMC (Fig. 8C). These results indicate that SMC induced endogenous IFN-β may act on EC and affect to the EC functions as well as autocrine regulation against SMC growth and apoptosis.

In this study, it was clarified that IFN-β enhances SMC apoptosis and inhibits the EC apoptosis and stimulates the EC growth. These effects were considered to be added as the mechanisms of the efficacy of IFN-β against hyperplastic arterial diseases. It was interested in examining more precisely the molecular mechanisms relating to differential effects of IFN-β on the apoptosis and growth of vascular SMC and EC.

## Abbreviations used

bFGF: basic fibroblast growth factor
CMV: cytomegaro virus
EDTA: ethylenediaminetetraacetic acid
ELISA: enzyme-linked immunosorbent assay
FCS: fetal calf serum
HAEC: human aortic endothelial cell
HCASMC: human coronary arterial smooth muscle cell
IFN: interferon
IL-1: interleukin-1
PBS: phosphate buffered saline
PDGF: platelet derived growth factor
PI: propidium iodide
pNA: p-nitrianiline
Poly I/C: polyriboinosinic-ribocytidylic acid
RT-PCR: reverse transcription polymerase chain reaction
SSCP: single-strand conformation polymorphism
TNF-α: tumor necrosis factor-α
VEGF: vascular endothelial growth factor
